# Emergence of *Morganellaceae* Harboring *bla*_IMP-27_ Metalloenzyme in Canada

**DOI:** 10.1128/mSphere.00048-21

**Published:** 2021-05-19

**Authors:** David A. Boyd, Laura F. Mataseje, Tanis Dingle, Linda Hoang, Brigitte Lefebvre, Allison McGeer, Roberto G. Melano, Ian Stuart, Andrew Walkty, Amanda Wilmer, Michael R. Mulvey

**Affiliations:** a Antimicrobial Resistance & Nosocomial Infections, National Microbiology Laboratory, Public Health Agency of Canada, Winnipeg, Manitoba, Canada; b University of Alberta, Provincial Laboratory for Public Health, Edmonton, Alberta, Canada; c Bacteriology & Mycology, BCCDC Public Health Laboratory, Vancouver, British Columbia, Canada; d Laboratoire de Santé Publique du Québec, Institut National de Santé Publique du Québec, Sainte-Anne-de-Bellevue, Québec, Canada; e Department of Microbiology, Mount Sinai Hospital, Toronto, Ontario, Canada; f Public Health Ontario Laboratories, Toronto, Ontario, Canada; g Horizon Health Network, Fredricton, New Brunswick, Canada; h Departments of Medical Microbiology and Infectious Diseases, Max Rady College of Medicine, University of Manitoba, Winnipeg, Manitoba, Canada; i Department of Laboratory Medicine, Kelowna General Hospital, Kelowna, British Columbia, Canada; Antimicrobial Development Specialists, LLC

**Keywords:** *Morganellaceae*, antimicrobial resistance, metallo-beta-lactamase

## Abstract

In 2018 to 2019, PCR for carbapenemases in routine Gram-negative isolates submitted to the National Microbiology Laboratory revealed an increase in IMP-type metalloenzyme-positive isolates, mostly among *Morganellaceae*. Whole-genome sequencing revealed that 23 *Morganellaceae* harbored *bla*_IMP-27_ within a chromosomal Tn*7* element. Phylogenomics indicated diversity of isolates but also the presence of a few clonal isolates dispersed geographically. These isolates may be difficult to detect due to carbapenem susceptibility and false-negative results in phenotypic testing.

**IMPORTANCE** Over the last decade or so, the frequency of isolation of clinical carbapenemase-producing organisms (CPOs) has increased among health care-associated infections. This may seriously compromise antimicrobial therapy, as carbapenems are considered the last line of defense against these organisms. The ability of carbapenemases to hydrolyze most β-lactams in addition to the co-occurrence of mechanisms of resistance to other classes of antimicrobials in CPOs can leave few options for treating infections. The class B metalloenzymes are globally distributed carbapenemases, and the most commonly found include the NDM, VIM, and IMP types. Our study describes a sudden emergence of IMP-27-harboring *Morganellaceae* during 2018 to 2019 in Canada. There is a paucity of literature on IMP-27 isolates, and our data bolster the information on the genetic context, antimicrobial profiles, and phylogenomics of this group of CPOs.

## INTRODUCTION

Carbapenemases, β-lactamases that hydrolyze carbapenem β-lactams, have been found globally among clinically significant members of the *Enterobacterales* (e.g., Escherichia coli, Klebsiella spp., Enterobacter spp., and *Citrobacter* spp.) and *Pseudomonadales* (e.g., Pseudomonas aeruginosa and Acinetobacter spp.) (1). The most prevalent carbapenemases are the so-called “big 5,” namely, KPC (class A), NDM, VIM, IMP (class B metallo-β-lactamases), and OXA-48 (class D). Though they are internationally distributed, some enzyme groups tend to be more prevalent in specific countries or areas (1, 2). The class B IMP enzymes, though found worldwide, tend to be more successfully established in Southeast Asia and the South Pacific regions and have occurred only sporadically in North America (2). Currently, 73 variants of IMP have been assigned (https://www.ncbi.nlm.nih.gov/pathogens/beta-lactamase-data-resources/). In Canada, the first IMP carbapenemase identified was *bla*_IMP-7_ from an outbreak of nosocomial P. aeruginosa isolated from 1995 to 1997 in a single region (3). The Canadian Nosocomial Infections Surveillance Program identified only two IMP producers among 615 carbapenemase-producing *Enterobacterales* collected from 2010 to 2016, an Enterobacter cloacae isolate harboring *bla*_IMP-13_ and an Acinetobacter pittii isolate harboring *bla*_IMP-26_ (4, 5). IMP-27 was first reported in 2011 from Proteus mirabilis PM185, isolated in 2009, with further studies determining that *bla*_IMP-27_ was on the chromosome in PM185, on an IncX8 plasmid and the chromosome in P. mirabilis PM187, and on a plasmid of unknown Inc type isolated from Providencia rettgeri PR1 (6–8). P. mirabilis GN855 harboring *bla*_IMP-27_ was reported from a patient in Ontario, Canada, in 2012 (9). Another study reported *bla*_IMP-27_ located on an IncQ1 plasmid found in multiple species of *Enterobacterales*, including P. mirabilis, Morganella morganii, and *P. rettgeri*, isolated from the environment of a swine operation in the United States (10).

## RESULTS

### Bacteria harboring *bla*_IMP-27_.

In 2018 and 2019 the National Microbiology Laboratory (NML) screened 2270 Gram-negative isolates by PCR for the most common carbapenemase gene groups, KPC, OXA-48, NDM, VIM, IMP, GES, and NMC/IMI. Twenty-eight isolates (1.2%) were positive by PCR for a *bla*_IMP_ gene, including one *P. rettgeri*, 15 P. mirabilis, seven M. morganii, and five P. aeruginosa isolates. In 2017, of 242 P. aeruginosa and 30 *Morganellaceae* isolates received for routine carbapenemase PCR, four P. aeruginosa isolates and one M. morganii isolate (N17-03220) harbored an IMP gene. M. morganii N17-03220 was later found to be indistinguishable by pulsed-field gel electrophoresis (PFGE) from M. morganii N18-00103 received 56 days later (January 2018) and in fact was from the same patient, and it was no longer studied. Thus, there was a significant increase of *Morganellaceae* harboring *bla*_IMP_ received by the NML after 2017. The 28 IMP-harboring isolates from 2018 to 2019 were from central (*n* = 9), western (*n* = 18), or eastern (*n* = 1) Canada and were isolated mainly from urine (*n* = 17), wounds (*n* = 4), or rectal swabs (*n* = 4). Whole-genome sequencing (WGS) analysis of all 2018–2019 isolates and P. mirabilis GN855 determined that all M. morganii isolates, 14 of the P. mirabilis isolates, and the *P. rettgeri* isolate harbored *bla*_IMP-27_, while among the P. aeruginosa isolates, one harbored *bla*_IMP-7_, one *bla*_IMP-62_, and three *bla*_IMP-26_. The *bla*_IMP-27_ gene could not be identified from the WGS data of one IMP PCR-positive P. mirabilis isolates and was presumed lost after subculture; therefore, this isolate was not further studied. Thus, among all the *Morganellaceae* received by the NML in 2018 to 2019 (*n* = 82) 26.8% (*n* = 22) were confirmed to harbor *bla*_IMP-27_.

### Antimicrobial susceptibility and detection of *bla*_IMP-27_-harboring isolates.

Antimicrobial susceptibilities were determined for all IMP-harboring isolates as well as a few non-carbapenemase-producing organisms (CPOs) for comparative purposes (Table 1). As expected for *Morganellaceae*, most were intermediate (I) or resistant (R) to imipenem regardless of the presence/absence of IMP-27, confirming that this is not a suitable phenotype for indicating the possible presence of a carbapenemase. Gradient diffusion was poor for indicating IMP-27 presence, as most isolates were susceptible (S) to meropenem and ertapenem. By Sensititre testing, all IMP-27 P. mirabilis isolates and the *P. rettgeri* isolate were I or R to all carbapenems, while the non-CPOs were S to the three nonimipenem carbapenems. However, all of the M. morganii isolates were S to all nonimipenem carbapenems by Sensititre testing.

**TABLE 1:**
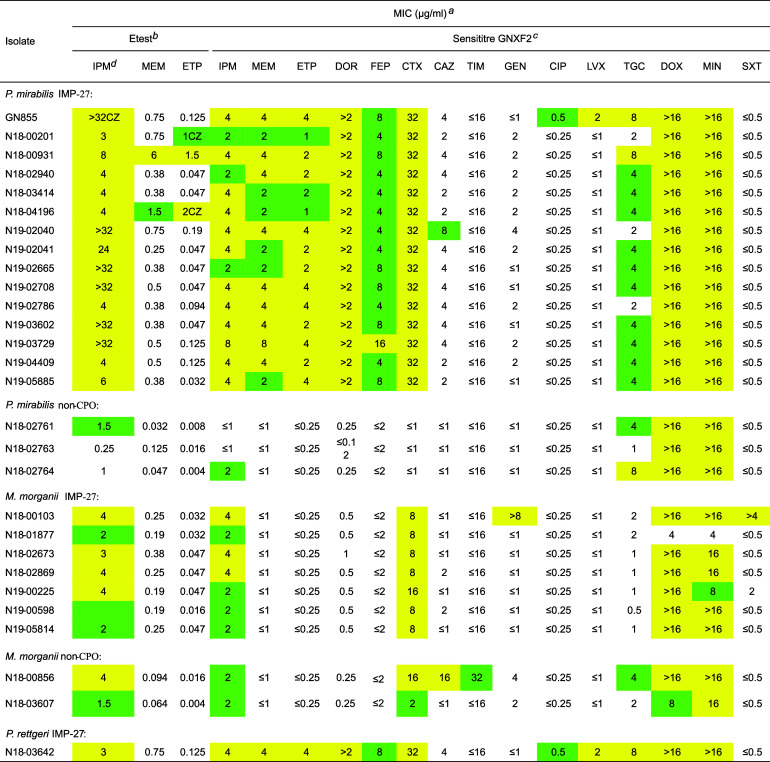
Antimicrobial susceptibilities of the isolates in this study

a Cell color indicates antimicrobial susceptibility category: yellow indicates resistance, green indicates intermediate or dose-dependent susceptibility (cefepime), and no color indicates susceptibility.

b Etest values are as read, but for categorization, they are rounded up to the nearest doubling dilution. CZ, colonies in the zone.

c Aztreonam, piperacillin-tazobactam, amikacin, and tobramycin are not listed as all isolates were susceptible.

d Abbreviations: AMK, amikacin; CAZ, ceftazidime; CIP, ciprofloxacin; CTX, cefotaxime; DOR, doripenem; DOX, doxycycline; ETP, ertapenem; FEP, cefepime; GEN, gentamicin; IPM, imipenem; LVX, levofloxacin; MEM, meropenem; MIN, minocycline; TGC, tigecycline; TIM, ticarcillin-clavulanate; TOB, tobramycin; TZP, piperacillin-tazobactam; SXT, sulfamethoxazole-trimethoprim.

Full antibiograms were in congruence with the resistomes (Table 2). Among the phenotypic tests (Table 2), the modified carbapenem inactivation method (mCIM) test was 100% specific and sensitive for carbapenemase presence/absence. All mCIM-positive isolates were also positive by EDTA-modified CIM (eCIM), correctly indicating the presence of a class B enzyme. The β-Carba test was 100% sensitive and specific for M. morganii and *P. rettgeri*, but all IMP-27-producing P. mirabilis isolates were falsely negative. The Carba-NP and Neo-Rapid Carb test, which work on the same principle, performed poorly, and all IMP-27-producing P. mirabilis isolates and the *P. rettgeri* isolate were falsely negative. Among IMP-27 M. morganii isolates, results for the Carba-NP and Neo-Rapid Carb tests were variable, with some exhibiting false-negative, invalid, or weakly positive results.

**TABLE 2:** Resistome, plasmid types, and results of phenotypic tests for carbapenemase activity for the isolates in this study^*a*^

Isolate	Resistome^*b*^	Plasmid type	Phenotypic test for carbapenemase activity^*c*^				
			mCIM^*d*^	**β**-Carba	Carba-NP	Neo-Rapid Carb	NG-Test CARBA 5^*e*^
P. mirabilis IMP-27							
GN855	*bla*_IMP-27_, *aadA*1, *cat*, *tet*(J)	No hits	POS	NEG	NEG	NEG	NEG
N18-00201	*bla*_IMP-27_, *aadA*1, *cat*, *tet*(J)	No hits	POS	NEG	NEG	NEG	NEG
N18-00931	*bla*_IMP-27_, *aadA*1, *cat*, *tet*(J)	No hits	POS	NEG	NEG	NEG	Not done
N18-02940	*bla*_IMP-27_, *aadA*1, *cat*, *tet*(J)	No hits	POS	NEG	NEG	NEG	Not done
N18-03414	*bla*_IMP-27_, *aadA*1, *cat*, *tet*(J)	No hits	POS	NEG	NEG	NEG	Not done
N18-04196	*bla*_IMP-27_, *aadA*1, *cat*, *tet*(J)	No hits	POS	NEG	NEG	NEG	Not done
N19-02040	*bla*_IMP-27_, *aadA*1, *cat*, *tet*(J)	No hits	POS	NEG	NEG	NEG	Not done
N19-02041	*bla*_IMP-27_, *aadA*1, *cat*, *tet*(J)	No hits	POS	NEG	NEG	NEG	Not done
N19-02665	*bla*_IMP-27_, *aadA*1, *cat*, *tet*(J)	No hits	POS	NEG	NEG	NEG	Not done
N19-02708	*bla*_IMP-27_, *aadA*1, *cat*, *tet*(J)	No hits	POS	NEG	NEG	NEG	Not done
N19-02786	*bla*_IMP-27_, *aadA*1, *cat*, *tet*(J)	No hits	POS	NEG	NEG	NEG	Not done
N19-03602	*bla*_IMP-27_, *aadA*1, *cat*, *tet*(J)	No hits	POS	NEG	NEG	NEG	Not done
N19-03729	*bla*_IMP-27_, *aadA*1, *cat*, *tet*(J)	No hits	POS	NEG	NEG	NEG	Not done
N19-04409	*bla*_IMP-27_, *aadA*1, *cat*, *tet*(J)	No hits	POS	NEG	NEG	NEG	Not done
N19-05885	*bla*_IMP-27_, *aadA*1, *cat*, *tet*(J)	No hits	POS	NEG	NEG	NEG	Not done

P. mirabilis non-CPO							
N19-02761	*cat*, *tet*(J)	No hits	NEG	NEG	NEG	NEG	NEG
N19-02763	*cat*, *tet*(J)	No hits	NEG	NEG	NEG	NEG	Not done
N19-02764	*cat*, *tet*(J)	No hits	NEG	NEG	NEG	NEG	Not done

M. morganii IMP-27							
N18-00103	*bla*_IMP-27_, *bla*_DHA-14_, *bla*_TEM-1B_, *aadA1*, *aadA2*, *aac(3)*-*Iid*, *aph(6)Id*, *aph(3′′)Ib*, *aph(3′)Ia*, *mph(A)*, *catA1*, *tet*(B), *sul1*, *sul2*, *dfrA12*	IncQ1	POS	POS	Invalid	wPOS	wPOS
N18-01877	*bla*_IMP-27_, *bla*_DHA-1_, *aadA1*, *catA2*	ColI (RGK), ColI440I	POS	POS	Invalid	POS	Not done
N18-02673	*bla*_IMP-27_, *bla*_DHA-1_, *aadA1*, *catA2*, *tet*(B)	No hits	POS	POS	NEG	wPOS	Not done
N18-02869	*bla*_IMP-27_, *bla*_DHA-1_, *aadA1*, *catA2*, *tet*(B)	No hits	POS	POS	NEG	wPOS	wPOS
N19-00225	*bla*_IMP-27_, *bla*_DHA-16_, *aadA1*, *catA2*, *tet*(D), *dfrA1*	No hits	POS	POS	NEG	wPOS	Not done
N19-00598	*bla*_IMP-27_, *bla*_DHA-1_, *aadA1*, *catA2*, *tet*(B)	No hits	POS	POS	POS	POS	Not done
N19-05814	*bla*_IMP-27_, *bla*_DHA-1_, *aadA1*, *catA2*, *tet*(B)	No hits	POS	POS	POS	POS	Not done

M. morganii non-CPO							
N18-00856	*bla*_DHA-1_, *tet*(B), *catA2*	No hits	NEG	NEG	NEG	NEG	Not done
N18-03607	*bla*_DHA-1_, *tet*(B), *catA2*	IncX2, *repA* (FII)	NEG	NEG	NEG	NEG	NEG

*P. rettgeri* IMP-27:							
N18-03642	*bla*_IMP-27_, *aadA1*	No hits	POS	POS	POS	NEG	Not done

a Resistome and plasmid types were determined by ResFinder and PlasmidFinder, respectively.

b The *sat-2* gene was not in the ResFinder database. The *bla*_DHA_ gene is the intrinsic *ampC* gene of M. morganii.

c POS, positive; NEG, negative. “Invalid” means that the no-meropenem control turned orange-yellow. “wPOS” means that an orange color was observed for the Carba-NP or NeoRapid Carb test or that a faint IMP band was observed in the NG-Test CARBA 5 test.

d All mCIM-positive isolates were also positive in the eCIM test.

e Immunochromatographic assay to detect KPC, OXA-48-like, VIM, IMP, and NDM enzymes.

We also tried the more expensive NG-Test CARBA 5 immunochromatographic assay on a small number of isolates, even though the package insert (ENO022CAR/Rev: 200131) does not list IMP-27 as one of the variants that can be detected by this test (Table 2). When the cells were obtained from tryptic soy agar (TSA)-blood plates (M. morganii) or Mueller-Hinton medium (P. mirabilis), all results were negative. Upon repeat testing with cells obtained from Mueller-Hinton containing 100 μg/ml ampicillin, a faint IMP-specific band was observed for the two IMP-27-harboring M. morganii isolates, though it was observed 5 to 10 min after the recommended test time of 15 min. The mCIM results indicate that IMP-27 is produced by all the *bla*_IMP-27_-harboring isolates in the study. Nonetheless, we determined specific activity against imipenem for the isolates tested by NG-Test CARBA 5 and confirmed imipenemase activity in the IMP-27-harboring isolates, though the activities can vary by 2- to 5-fold (Table 3).

**TABLE 3:** Specific activities of crude lysates against imipenem from some isolates in this study

Isolate	Sp act (μmol min^−1^ mg^−1^)
P. mirabilis	
GN 855 (IMP-27)	70.1 ± 19.6
N18-00201 (IMP-27)	28.0 ± 5.1
N18-02761 (non-CPO)	None detected
M. morganii	
N18-00103 (IMP-27)	149.7 ± 22.1
N18-02869 (IMP-27)	70.7 ± 5.8
N18-03607 (non-CPO)	None detected

Together, the results indicate that P. mirabilis is likely recalcitrant to lysis/permeabilization in the non-mCIM phenotypic tests, all of which have a cell suspension/lysis solution. For the M. morganii isolates, although results indicate that some lysis does occur, it may be suboptimal, and this, combined with low IMP-27 levels for some isolates and/or technical issues, may account for poor results in the non-mCIM phenotypic tests.

### *bla*_IMP-27_ is found within a Tn*7* element located in the chromosome.

**FIG 1:**
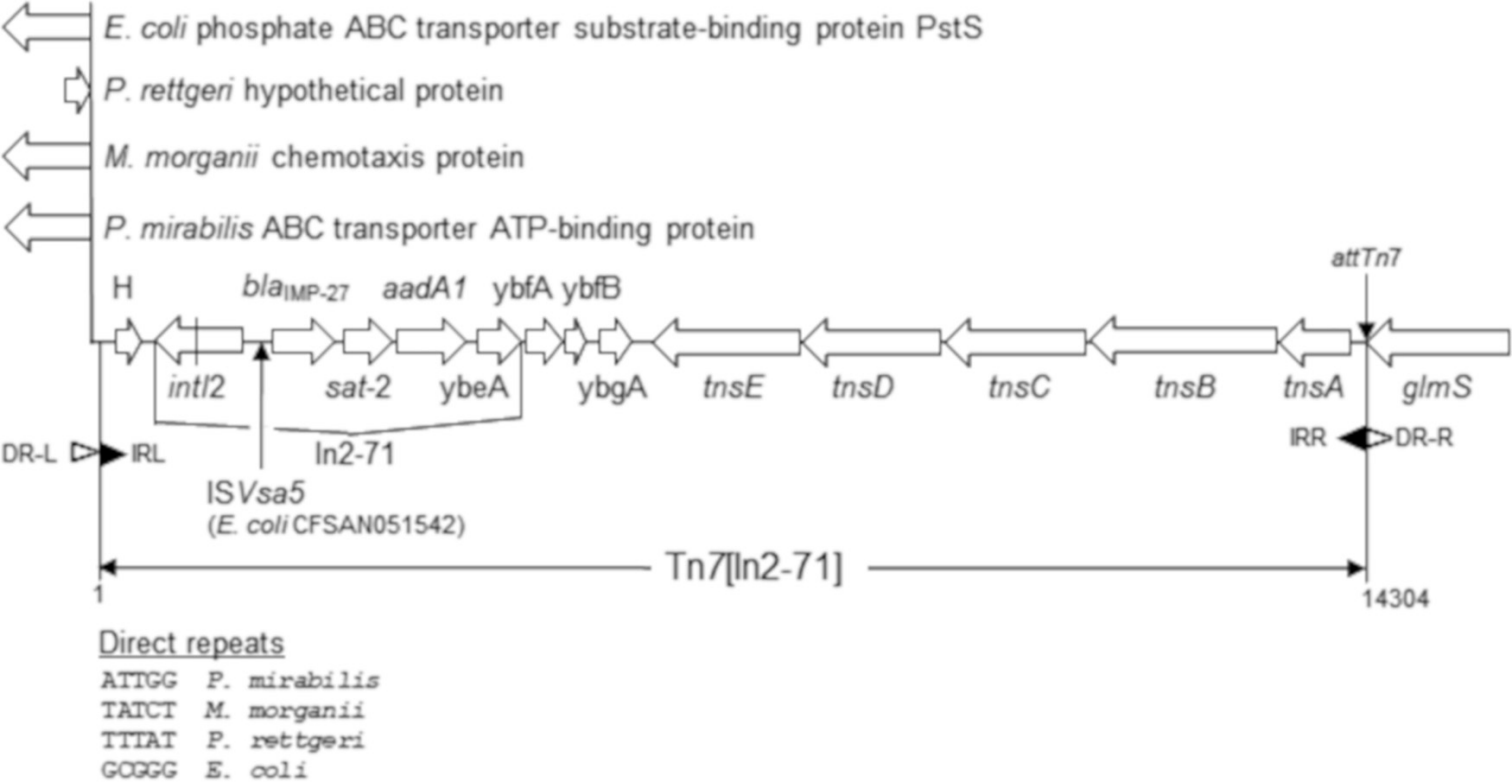
Schematic diagram depicting Tn*7*[In2-71] and its position in the chromosome. The Tn*7*[In2-71] element in E. coli CFSAN051542 is 15,642 bp, as it harbors an ISVsa5 element between the *intI2* and *bla*_IMP-27_ genes. The *intI2* gene contains an internal stop codon, indicated by a vertical line. The coordinates for Tn*7*[In2-71] in the genomes are as follows: E. coli CFSAN05142, 4844260 to 4859901 (accession no. CP020835); *P. rettgeri* N18-03642, 27829 to 42132 (accession no. JAAOIA010000015); M. morganii N18-00103, 19322 to 33625 (accession no. CP048275); P. mirabilis N18-00201, 3732335 to 3746638 (accession no. CP048404).

**FIG 2:**
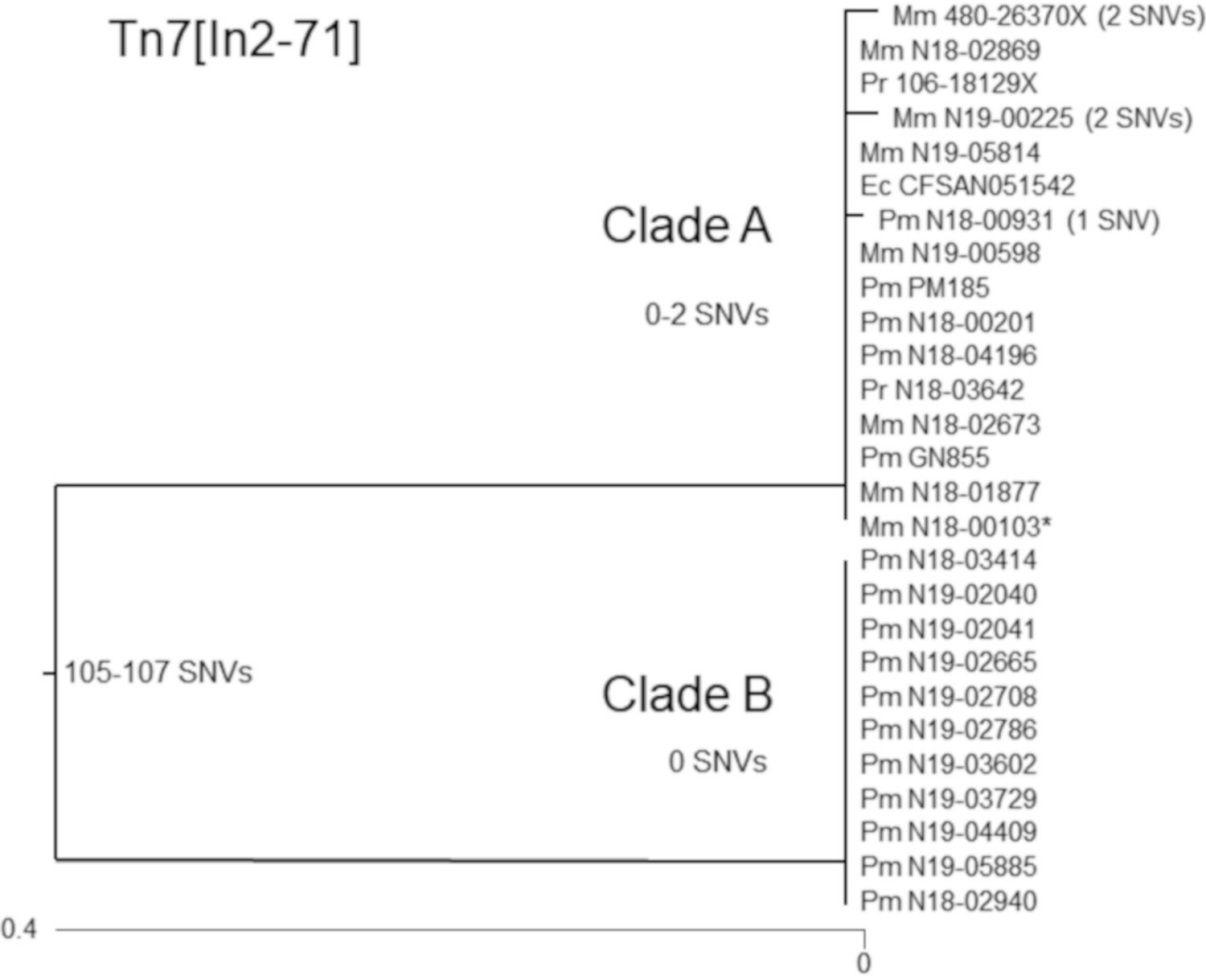
Phylogenetic tree of Tn*7*[In2-71] elements based on a multiple-sequence alignment. The SNV differences are from using the Tn*7*[In2-71] from M. morganii N18-00103 as the reference (indicated by an asterisk). The Tn*7*[In2-71] from E. coli CFSAN051542 was analyzed after removal of the IS*Vsa5* sequence and one of its target site duplications.

WGS analysis showed that the *bla*_IMP-27_ gene was located in the class 2 integron In2-71 (http://integrall.bio.ua.pt/?), which was integrated into a Tn*7* element (Fig. 1). This structure, labeled Tn*7*[In2-71], was inserted into the chromosome of all isolates via the *att*Tn*7* site at the 3′ end of the *glmS* gene, the canonical bacterial Tn*7* insertion site (11), and each element was flanked by direct repeats, indicating acquisition by transposition. Tn*7*[In2-71] elements were identified from the GenBank database (>99% identity) in P. mirabilis PM185 (accession no. NOWB01000038), *P. rettgeri* 106-1829X (accession no. KY847874), M. morganii 480-26370X (accession no. KY847873), and E. coli CFSAN051542 (accession no. CP020835). Sequence analysis divided the Tn*7*[In2-71] elements into two clades, A (*n* = 16) and B (*n* = 11), with the elements in clade A being >99.9% identical and the elements in clade B being 100% identical, but with the clades differing by 105 to 107 bp differences (Fig. 2). The vast majority of base pair differences were found in the *tnsA*-*tnsB* region indicating a region of recombination (data not shown). No plasmid replicons were identified in the P. mirabilis or the *P. rettgeri* isolates, whereas two M. morganii isolates harbored replicons (Table 2). Though IncQ1 plasmids have been found to harbor *bla*_IMP-27_ (10), the IncQ1 replicon in N18-00103 was found to be integrated into the chromosome and not linked to Tn*7*[In2-71].

### Limited clonality revealed by core genome SNV analysis.

**FIG 3:**
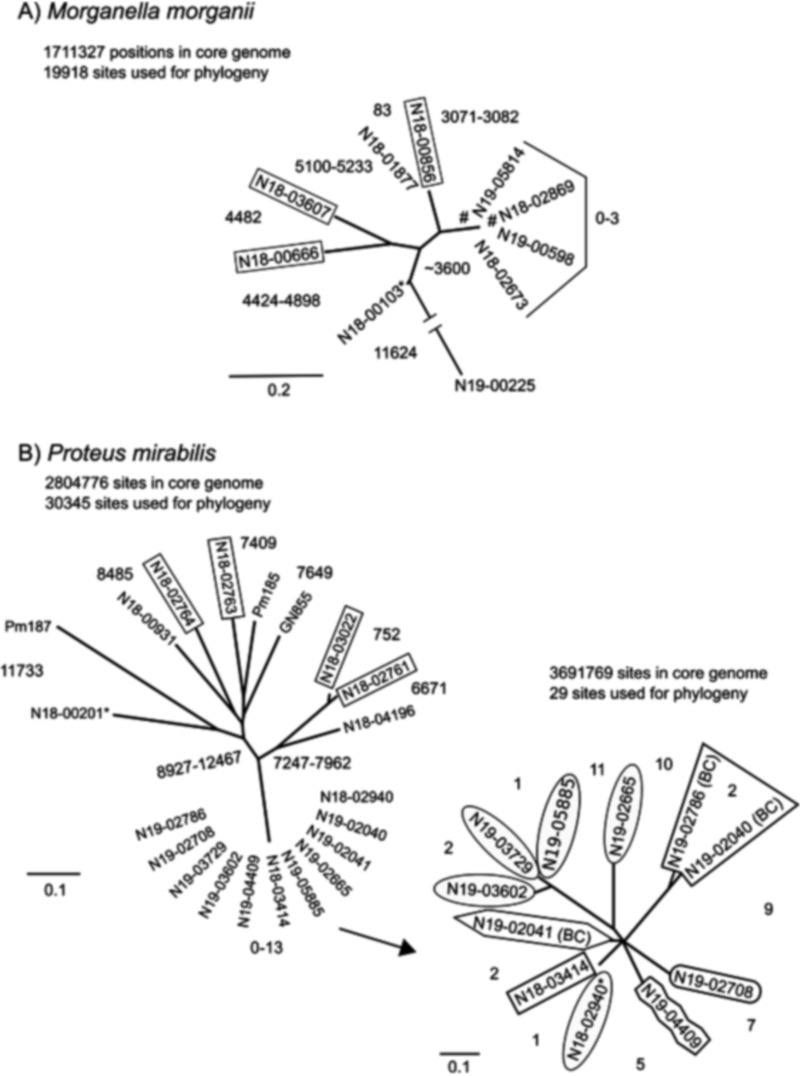
Phylogenetic trees of the (A) M. morganii and (B) P. mirabilis isolates in this study as generated by the SNVPhyl Pipeline, which generates an alignment of high-quality valid SNVs through PhyML using the GTR+γ model (15). Reference genomes used are indicated by an asterisk and were the closed genomes of M. morganii N18-00103 (CP048275) or P. mirabilis N18-00201 (CP048404) or a pseudogenome (concatenated contigs) of P. mirabilis N18-02940. SNVs or SNV ranges between isolates or groups of isolates are shown. For the main analysis of each group of the same species, boxed isolates do not harbor *bla*_IMP-27_. For the subanalysis of the cluster of the closely related P. mirabilis isolates, each unique shape indicates a specific facility from which the bacterium was isolated. The isolates were isolated in Alberta except for the three from British Columbia (BC).

We carried out core genome SNV analysis on all P. mirabilis and all M. morganii to determine strain relatedness (Fig. 3A and B). Among the M. morganii isolates, 6 of 10 isolates are diverse, with the number of single nucleotide variants (SNVs) between them ranging from 83 to >14,000 (Fig. 3A). Four isolates clustered at 0 to 3 SNVs, but no strong epidemiological links could be uncovered between any of the four patients, though two isolates were from patients who had been in the same hospital but 470 days apart. The analysis of P. mirabilis, which included the U.S. IMP-27 isolates PM185 and PM187, showed that 10 isolates were diverse, differing by 752 to >12,800 SNVs from each other (Fig. 3B). However, the 11 P. mirabilis isolates harboring Tn*7*[In2-71]-B (Fig. 2) clustered together at 0 to 13 SNVs or 1 to 15 SNVs when reanalyzed separately with an internal reference and, hence, a larger core genome. Anonymized patient facilities were available for some isolates, indicating some common facilities, but the limited data make inferring direct transmission events unfeasible. Nonetheless, this cluster of closely related isolates can be postulated to have derived from a common ancestor that has spread to multiple locations in western Canada.

## DISCUSSION

*Morganellaceae* isolates harboring *bla*_IMP-27_ have emerged in Canada since 2018. These isolates may be difficult to detect as CPOs, as they can exhibit susceptibility to carbapenems depending on which susceptibility testing method is used. The mCIM detected carbapenemase production or lack thereof among all study isolates, as did the β-Carba test for the M. morganii and *P. rettgeri* isolates. Though *bla*_IMP-27_ was exclusively chromosomally located here, its dissemination may be facilitated by being harbored within a mobile Tn*7* transposon. Isolates were diverse but phylogenomics revealed clones harboring *bla*_IMP-27_ have dispersed in Canada. The major limitation of this study was that isolates were voluntarily submitted to the NML, and thus, the prevalence of *bla*_IMP-27_ isolates may be underestimated. In addition, due to *bla*_IMP_ family sequence variation, in-house primers and some commercial assays may yield false-negative results (12).

## MATERIALS AND METHODS

### Bacterial isolates.

The bacteria in this study were from routine isolates voluntarily sent to the NML for carbapenemase PCR. Typically, organisms are sent because of a suspicion of carbapenemase production due to reduced susceptibility/resistance to a carbapenem and/or a positive result of a phenotypic method that indicates carbapenemase production. For the isolates that test positive, the PCR results are reported, and the carbapenemase gene is not sequenced unless by special request.

### Antimicrobial susceptibilities and phenotypic carbapenemase detection.

Antimicrobial susceptibilities were carried out by Etest (bioMérieux) and Sensititre GNX2F plates (Thermo Fisher Scientific, Toronto, ON, Canada). Categorical interpretations were done using CLSI (13) or FDA guidelines (tigecycline). The β-Carba test (Bio-Rad Laboratories, Mississauga, ON, Canada), Neo-Rapid Carb test (Roscoe Diagnostica, Taastrup, Denmark), and NG-Test CARBA 5 (NG Biotech, Guipry, France) were carried out per the manufacturer’s instructions. The Carba-NP, mCIM, and eCIM tests were carried out as described elsewhere (13).

### Carbapenemase multiplex PCR.

The carbapenemase multiplex PCR was as previously described (4) except with two updated primers, IMP-F2 (5′-CTTGAMGARGGYGTTTATGTTCATAC), which pairs with IMP-2, and IMI-Dr (5′-TCATTTGCMGTACCGTATGC), which pairs with IMI-A.

### Sequencing and bioinformatics.

Whole-genome sequencing (WGS) was carried out on all isolates by NextSeq (Illumina Inc., San Diego, CA), with two isolates (M. morganii N18-00103 and P. mirabilis N18-00201) additionally sequenced by Nanopore technology (Oxford Nanopore Technologies, Oxford, UK). Read assembly was carried out using Unicycler v0.4.4 (14). Single nucleotide variant (SNV) analysis was carried out using the SNVPhyl Pipeline (15). Assemblies were analyzed by the ResFinder and PlasmidFinder tools at the Center of Genomic Epidemiology website (http://www.genomicepidemiology.org).

### Data availability.

Nucleotide sequences and WGS reads have been deposited in NCBI BioProject PRJNA603518. The complete closed genomes of P. mirabilis N18-00201 and M. morganii N18-00103 and the draft genome of *P. rettgeri* N18-02642 have been assigned accession no. CP048404, CP048275, and JAAOIA000000000, respectively. The sequence of Tn*7*[In2-71] from N18-02940 has been assigned accession no. MT226801.

## References

[1] Nordmann P, Poirel L. 2014. The difficult-to-control spread of carbapenemase producers among Enterobacteriaceae worldwide. Clin Microbiol Infect 20:821–830. doi:10.1111/1469-0691.12719. 24930781

[2] Matsumura Y, Peirano G, Motyl MR, Adams MD, Chen L, Kreiswirth B, DeVinney R, Pitout JD. 2017. Global molecular epidemiology of IMP-producing Enterobacteriaceae. Antimicrob Agents Chemother 61:e02729-16. doi:10.1128/AAC.02729-16. 28167555 PMC5365671

[3] Gibb AP, Tribuddharat C, Moore RA, Louie TJ, Krulicki W, Livermore DM, Palepou M-FI, Woodford N. 2002. Nosocomial outbreak of carbapenem-resistant *Pseudomonas aeruginosa* with a new *bla*_IMP_ allele, *bla*_IMP-7_. Antimicrob Agents Chemother 46:255–258. doi:10.1128/aac.46.1.255-258.2002. 11751148 PMC126979

[4] Mataseje LF, Abdesselam K, Vachon J, Mitchel R, Bryce E, Roscoe D, Boyd DA, Embree J, Katz K, Kibsey P, Simor AE, Taylor G, Turgeon N, Langley J, Gravel D, Amaratunga K, Mulvey MR. 2016. Results from the Canadian Nosocomial Infection Surveillance Program on Carbapenemase-Producing Enterobacteriaceae, 2010 to 2014. Antimicrob Agents Chemother 60:6787–6794. doi:10.1128/AAC.01359-16. 27600052 PMC5075087

[5] Boyd DA, Mataseje LF, Pelude L, Mitchell R, Bryce E, Roscoe D, Embree J, Katz K, Kibsey P, Lavallee C, Simor AE, Taylor G, Turgeon N, Langley JM, Amaratunga K, Mulvey MR, Wong A, McGeer A, Simor A, Lee B, Frenette C, Ellis C, Lavallee C, Mertz D, Bryce E, Henderson E, Taylor G, German G, Davis I, de Heer J, Minion J, Embree J, Langley J, Srigley J, Embil J, Vayalumkal J, Suh K, Katz K, Johnston L, Lefebvre M-A, John M, Blackburn MM, Bridger N, Turgeon N, Thampi N, Kibsey P, Stagg P, Richardson S, Hota S, Pelletier S, members of the Canadian Nosocomial Infection Surveillance Program, et al. 2019. Results from the Canadian Nosocomial Infection Surveillance Program for detection of carbapenemase-producing *Acinetobacter* spp. in Canadian hospitals, 2010–16. J Antimicrob Chemother 74:315–320. doi:10.1093/jac/dky416. 30312401

[6] Moulds NM, Thomson KS, Hanson ND. 2011. IMP-27, a novel metallo-β-lactamase (MBL) associated with a class II integron identified in an isolate of *Proteus mirabilis*, abstr C1-1212. Abstr 51st Intersci Conf Antimicrob Agents Chemother.

[7] Dixon N, Fowler RC, Yoshizumi A, Horlyama T, Ishii Y, Harrison L, Geyer CN, Moland ES, Thomson K, Hanson ND. 2016. IMP-27, a unique metallo-β-lactamase identified in geographically distinct isolates of *Proteus mirabilis*. Antimicrob Agents Chemother 60:6418–6421. doi:10.1128/AAC.02945-15. 27503648 PMC5038328

[8] Potter RF, Wallace MA, McMullen AR, Prusa J, Stallings CL, Burnham CAD, Dantas G. 2018. *bla*_IMP-27_ on transferable plasmids in *Proteus mirabilis* and *Providencia rettgeri*. Clin Microbiol Infect 24:1019.E5–1019.E8. doi:10.1016/j.cmi.2018.02.018. PMC6105362 29496594

[9] Tiget N, Siebert H, MacNeill M, Rawte P, Farrell DJ, Low DE, Patel SN, Melano RG. 2012. Detection of IMP-27 metallo-β-lactamase in *Proteus mirabilis*, ON, Canada, abstr C2-090. Abstr 52st Intersci Conf Antimicrob Agents Chemother.

[10] Mollenkopf DF, Stull JW, Mathys DA, Bowman AS, Feicht SM, Grooters SV, Daniels JB, Wittum TE. 2017. Carbapenemase-producing *Enterobacteriaceae* recovered from the environment of a swine farrow-to-finish operation in the United States. Antimicrob Agents Chemother 61:e01298-16. doi:10.1128/AAC.01298-16. PMC5278694 27919894

[11] Peters JE. 2014. Tn*7*. Microbiol Spectr 2:MDNA3-0010-2014. doi:10.1128/microbiolspec.MDNA3-0010-2014. 26104363

[12] Lowe CF, Matic N, Champagne S, Romney MG, Leung V, Ritchie G. 2020. The Brief Case: IMP, the uncommonly common carbapenemase. J Clin Microbiol 58:e01094-19. doi:10.1128/JCM.01094-19. PMC7098757 32213572

[13] Clinical and Laboratory Standards Institute. 2020. Performance standards for antimicrobial susceptibility testing: M100, 30th ed. CLSI, Wayne, PA, USA.

[14] Wick RR, Judd LM, Gorrie CL, Holt KE. 2017. Unicycler: resolving bacterial genome assemblies from short and long sequencing reads. PLoS Comput Biol 13:e1005595. doi:10.1371/journal.pcbi.1005595. 28594827 PMC5481147

[15] Petkau A, Mabon P, Sieffert C, Knox NC, Cabral J, Iskander M, Iskander M, Weedmark K, Zaheer R, Katz LS, Nadon C, Reimer A, Taboada E, Beiko RG, Hsiao W, Brinkman F, Graham M, Van Domselaar G. 2017. SNVPhyl: a single nucleotide variant phylogenomics pipeline for microbial genomic epidemiology. Microb Genom 3:e000116. doi:10.1099/mgen.0.000116. 29026651 PMC5628696

